# Supplementary figures and images for: Precise localization and dynamic distribution of Japanese encephalitis virus in the rain nuclei of infected mice
Source: PLoS Negl Trop Dis. 2021 Jun 21;15(6):e0008442. doi: 10.1371/journal.pntd.0008442 (PMC8216507; doi:10.1371/journal.pntd.0008442)

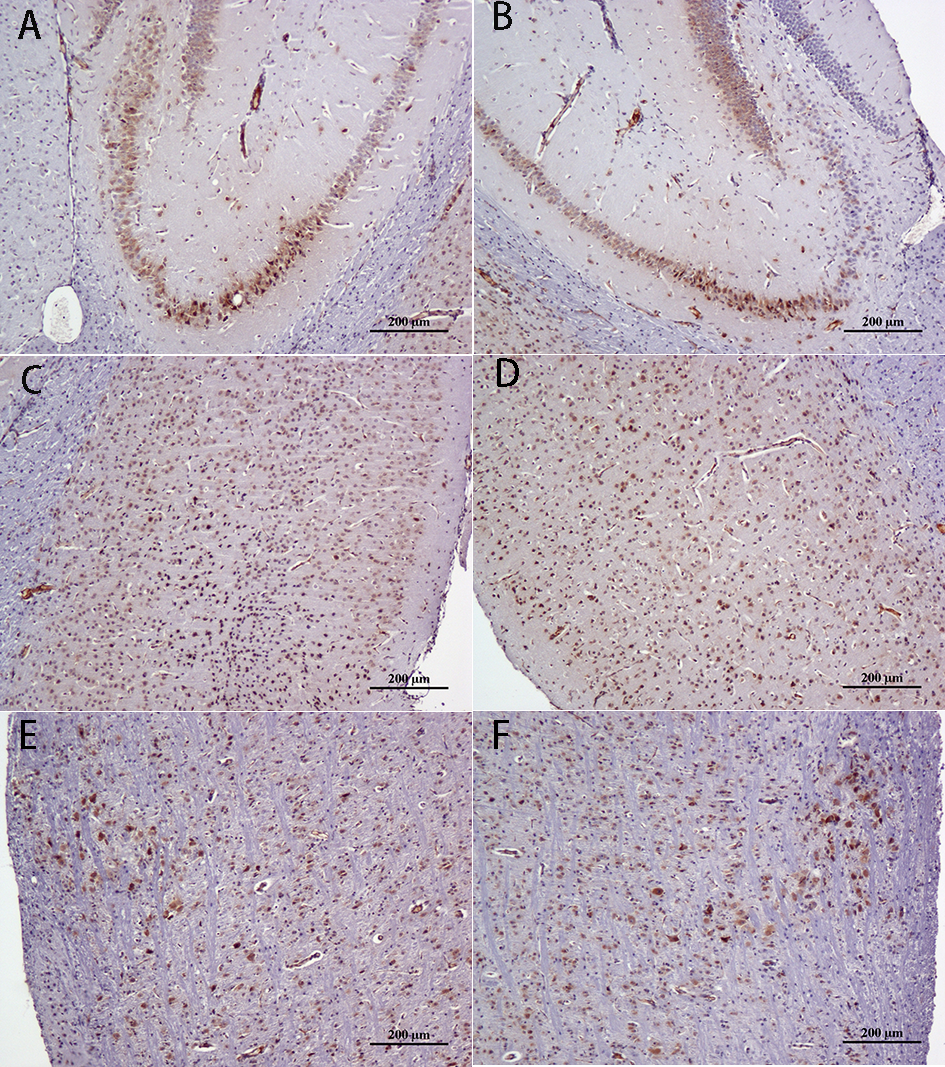

Supplement: S1 Fig — The same positive signals area appear in the three typical parts. Brain hippocampus A (left), B (right); cerebral cortex C (left), D (right) and medulla oblongata E (left), F (right). (IHC) (TIF) [file pntd.0008442.s001.tif]

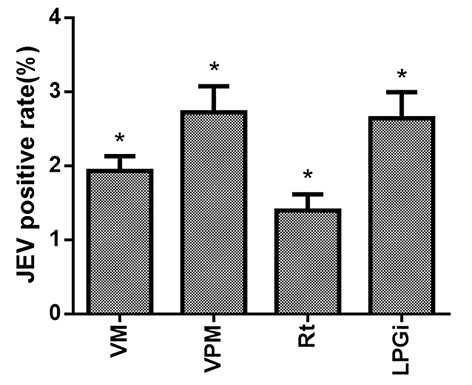

Supplement: S2 Fig — * P < 0.05, ** P < 0.01, n = 3. (TIF) [file pntd.0008442.s002.tif]

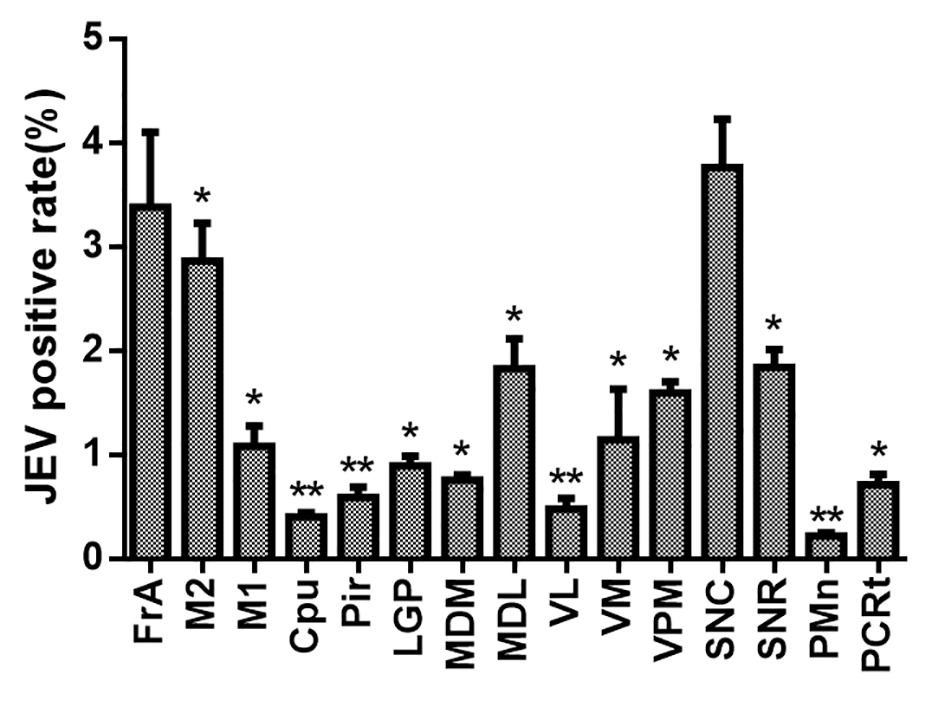

Supplement: S3 Fig — * P < 0.05, ** P < 0.01, n = 3. (TIF) [file pntd.0008442.s003.tif]

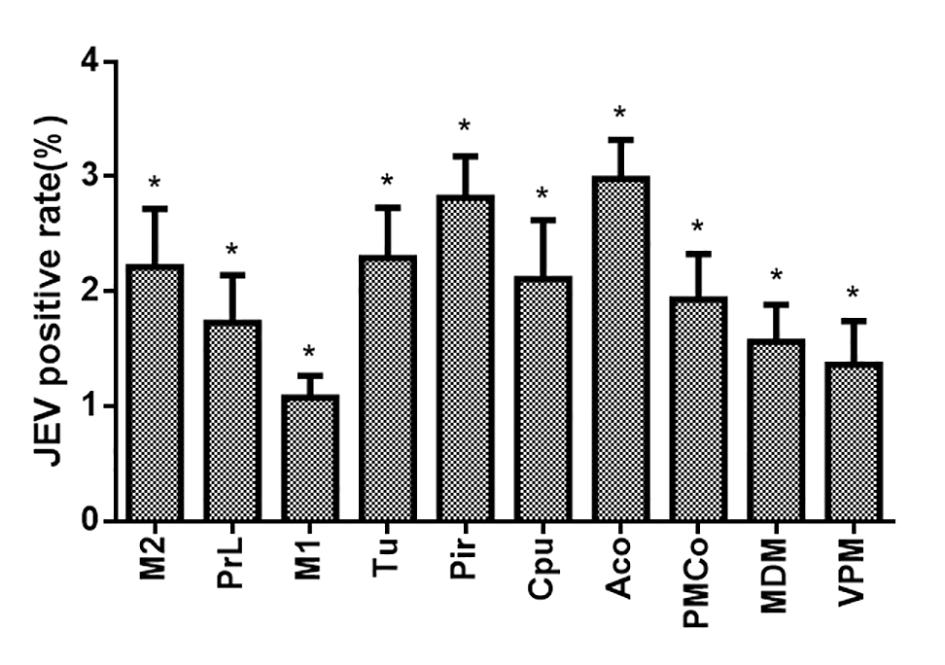

Supplement: S5 Fig — * P < 0.05, ** P < 0.01, n = 3. (TIF) [file pntd.0008442.s005.tif]

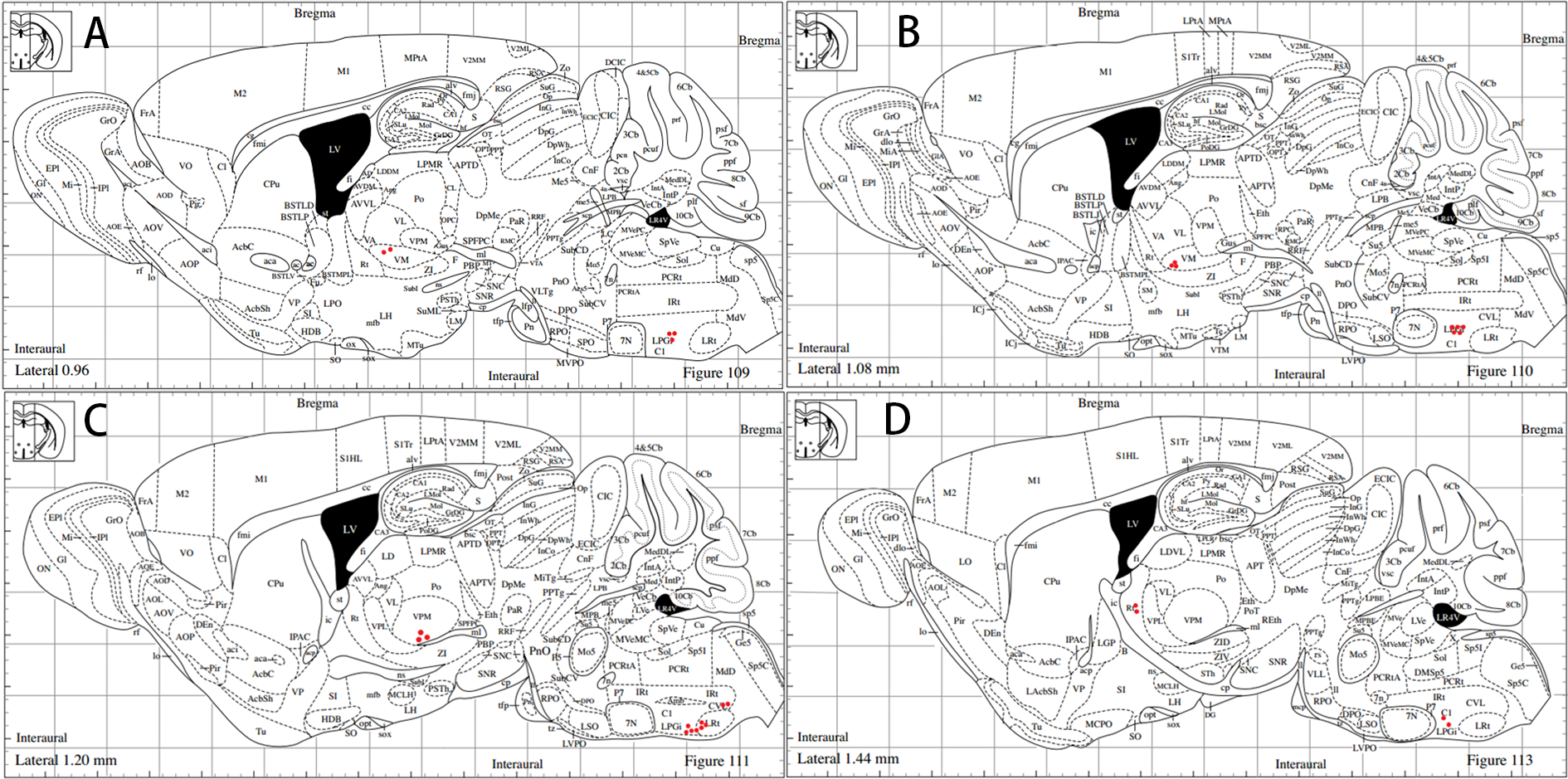

Supplement: S6 Fig — Plotted according to the reference literature [11], the red origin point represents the location of JEV distribution. Plotted according to the reference literature [11], the red origin point represents the location of JEV distribution. (A-D) Sagittal images of the brain at a distance of 0.96, 1.08, 1.20, and 1.44 mm from the mid-sagittal plane, respectively. (TIF) [file pntd.0008442.s006.tif]

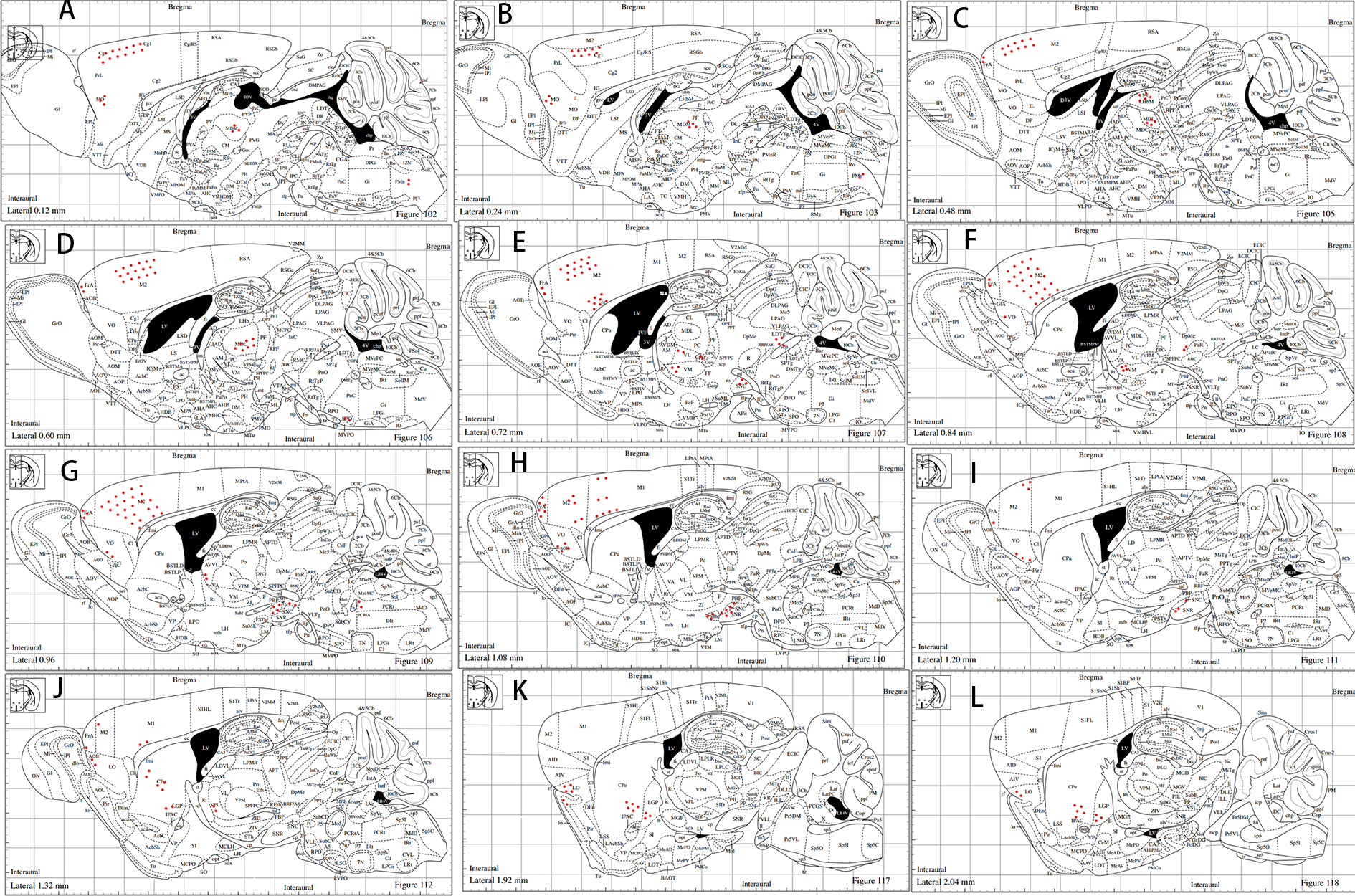

Supplement: S7 Fig — Plotted according to the reference literature [11], the red origin point represents the location of JEV distribution. (A-L) Sagittal images of the brain at a distance of 0.12, 0.24, 0.48, 0.60, 0.72, 0.84, 0.96, 1.08, 1.20, 1.32, 1.92, and 2.04 mm from the mid-sagittal plane, respectively. (TIF) [file pntd.0008442.s007.tif]

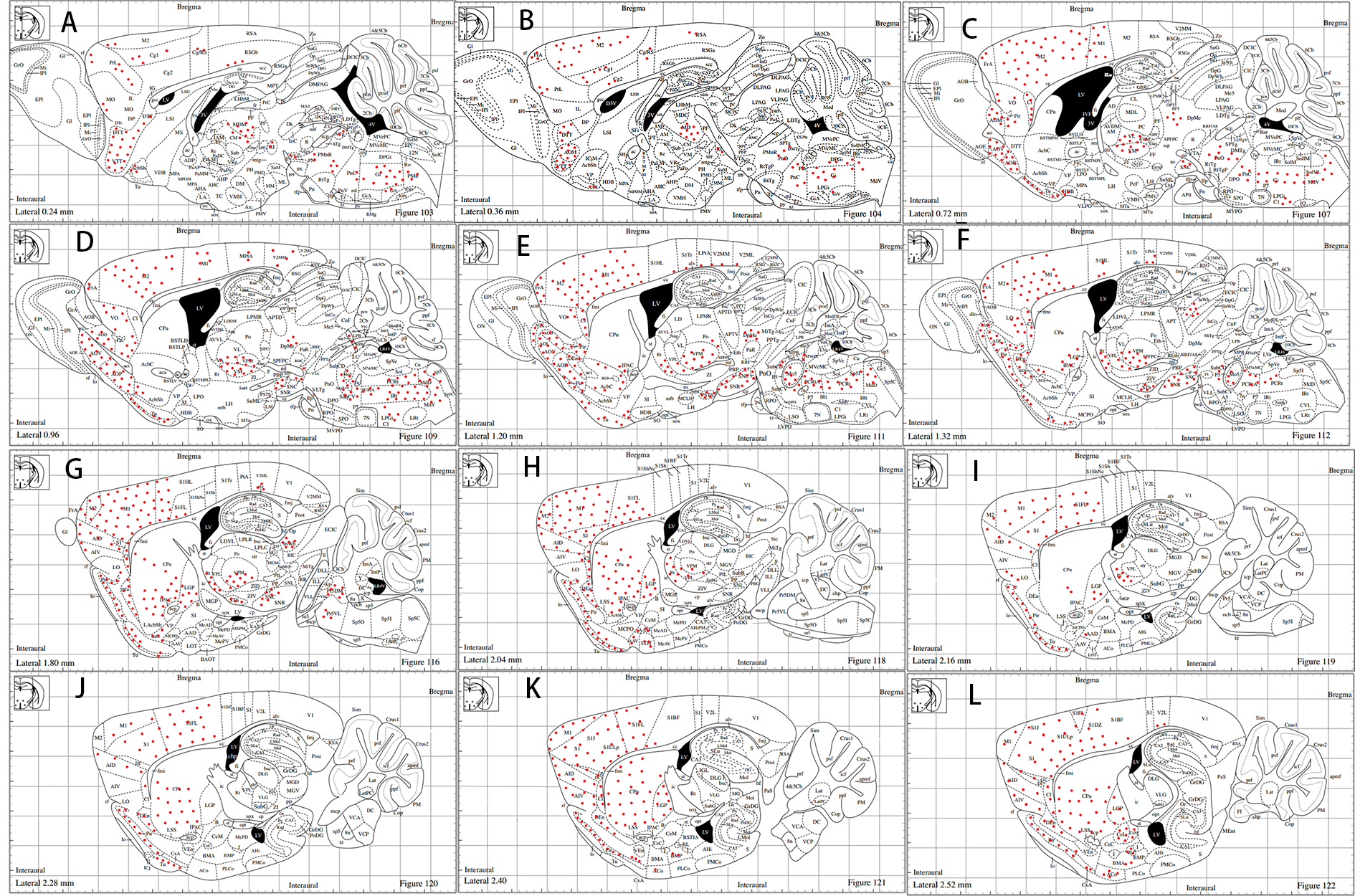

Supplement: S8 Fig — Plotted according to the reference literature [11], the red origin point represents the location of JEV distribution. (A-L) Sagittal images of the brain at a distance of 0.24, 0.36, 0.72, 0.96, 1.20, 1.32, 1.80, 2.04, and 2.16 mm from the mid-sagittal plane, respectively. (TIF) [file pntd.0008442.s008.tif]

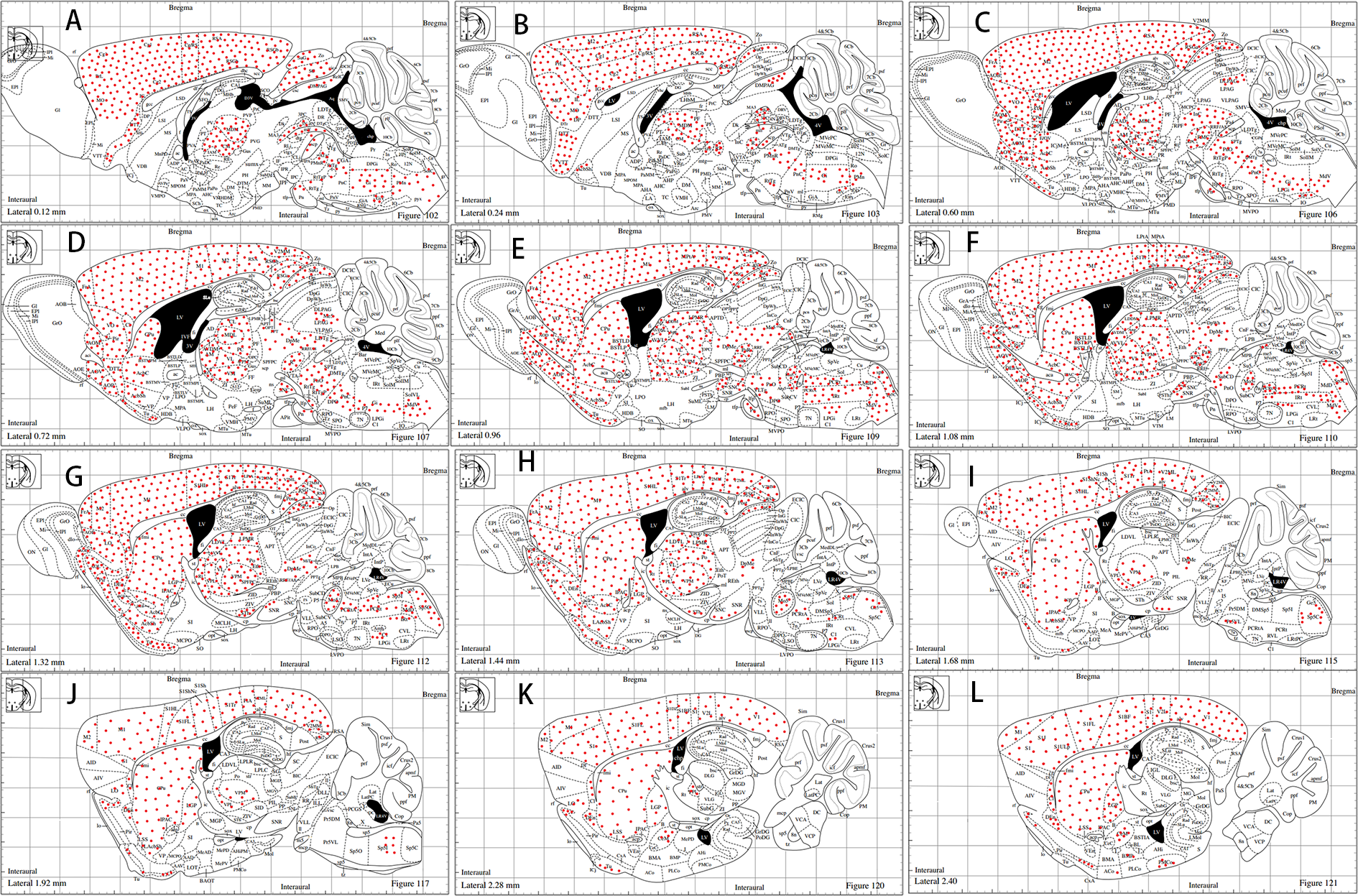

Supplement: S9 Fig — Plotted according to the reference literature [11], the red origin point represents the location of JEV distribution. (A-L) Sagittal images of the brain at a distance of 0.12, 0.24, 0.60, 0.72, 0.96, 1.08, 1.32, 1.44, 1.68, 1.92, 2.28, and 2.40 mm from the mid-sagittal plane, respectively. (TIF) [file pntd.0008442.s009.tif]

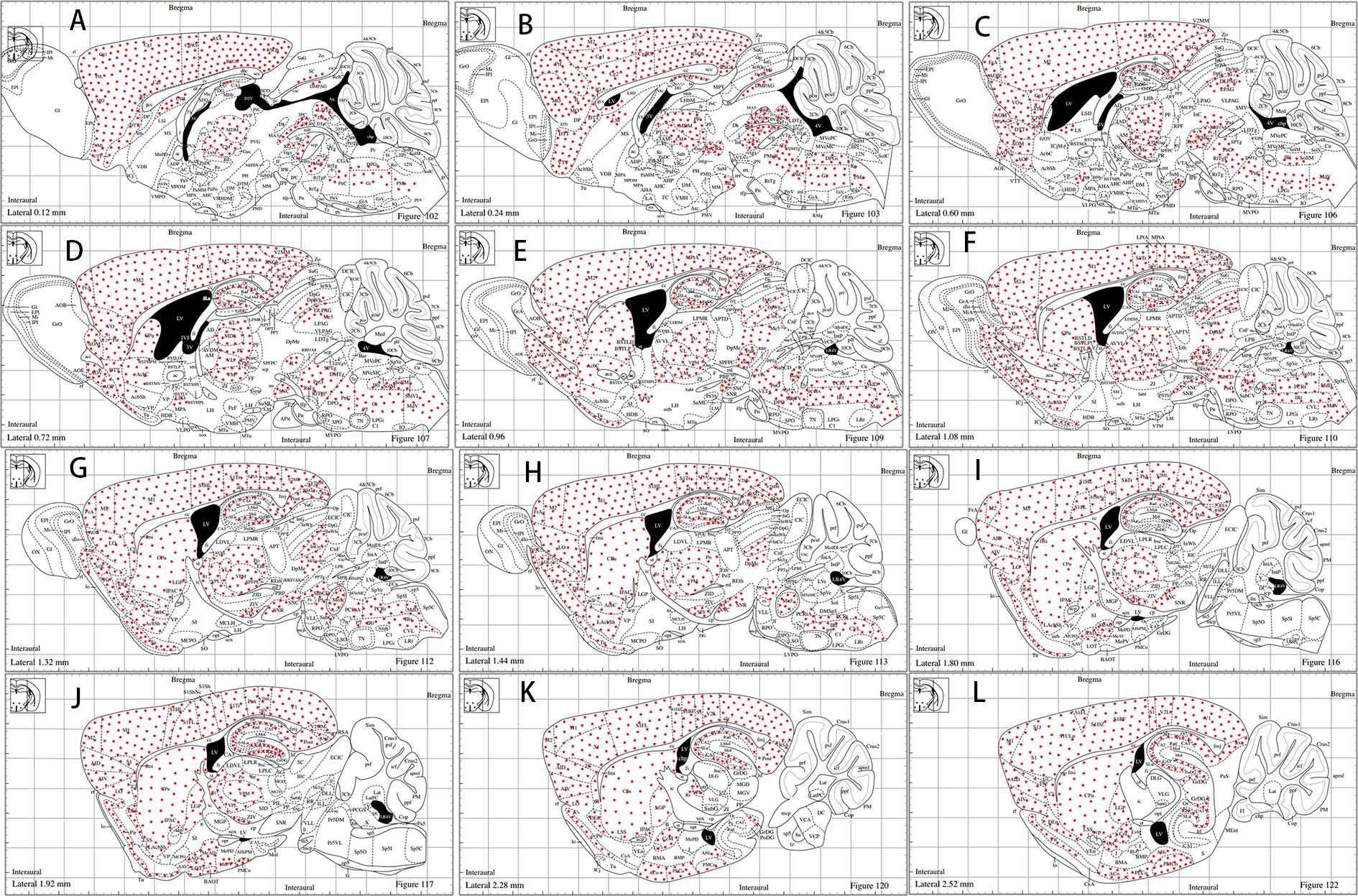

Supplement: S10 Fig — Plotted according to the reference literature [11], the red origin point represents the location of JEV distribution. (A-L) Sagittal images of the brain at a distance of 0.12, 0.24, 0.60, 0.72, 0.96, 1.08, 1.32, 1.44, 1.80, 1.92, 2.28, and 2.52 mm from the mid-sagittal plane, respectively. (TIF) [file pntd.0008442.s010.tif]

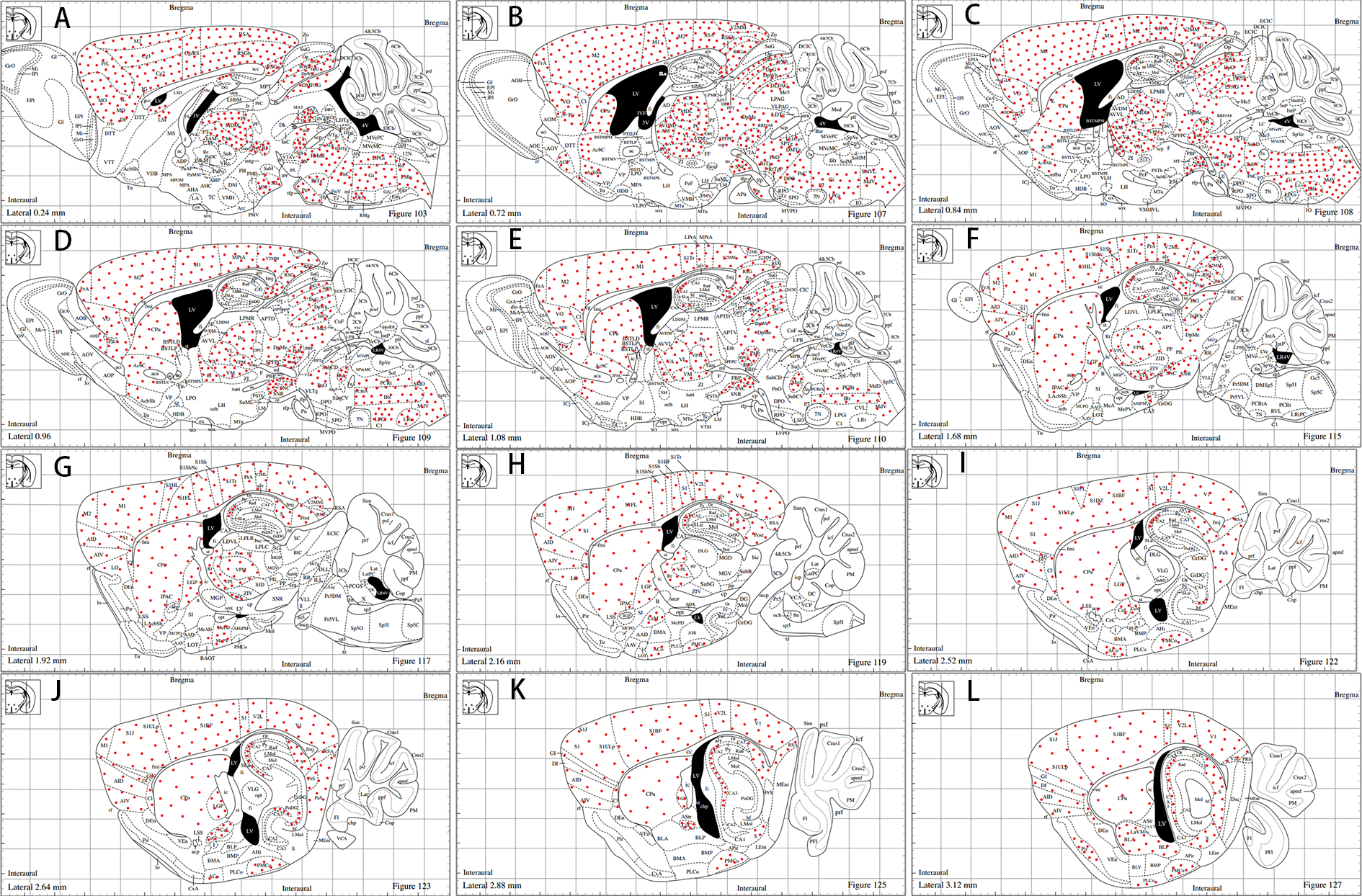

Supplement: S11 Fig — Plotted according to the reference literature [11], the red origin point represents the location of JEV distribution. (A-L) Sagittal images of the brain at a distance of 0.24, 0.72, 0.84, 0.96, 1.08, 1.68, 1.92, 2.16, 2.52, 2.64, 2.88, and 3.12 mm from the mid-sagittal plane, respectively. (TIF) [file pntd.0008442.s011.tif]

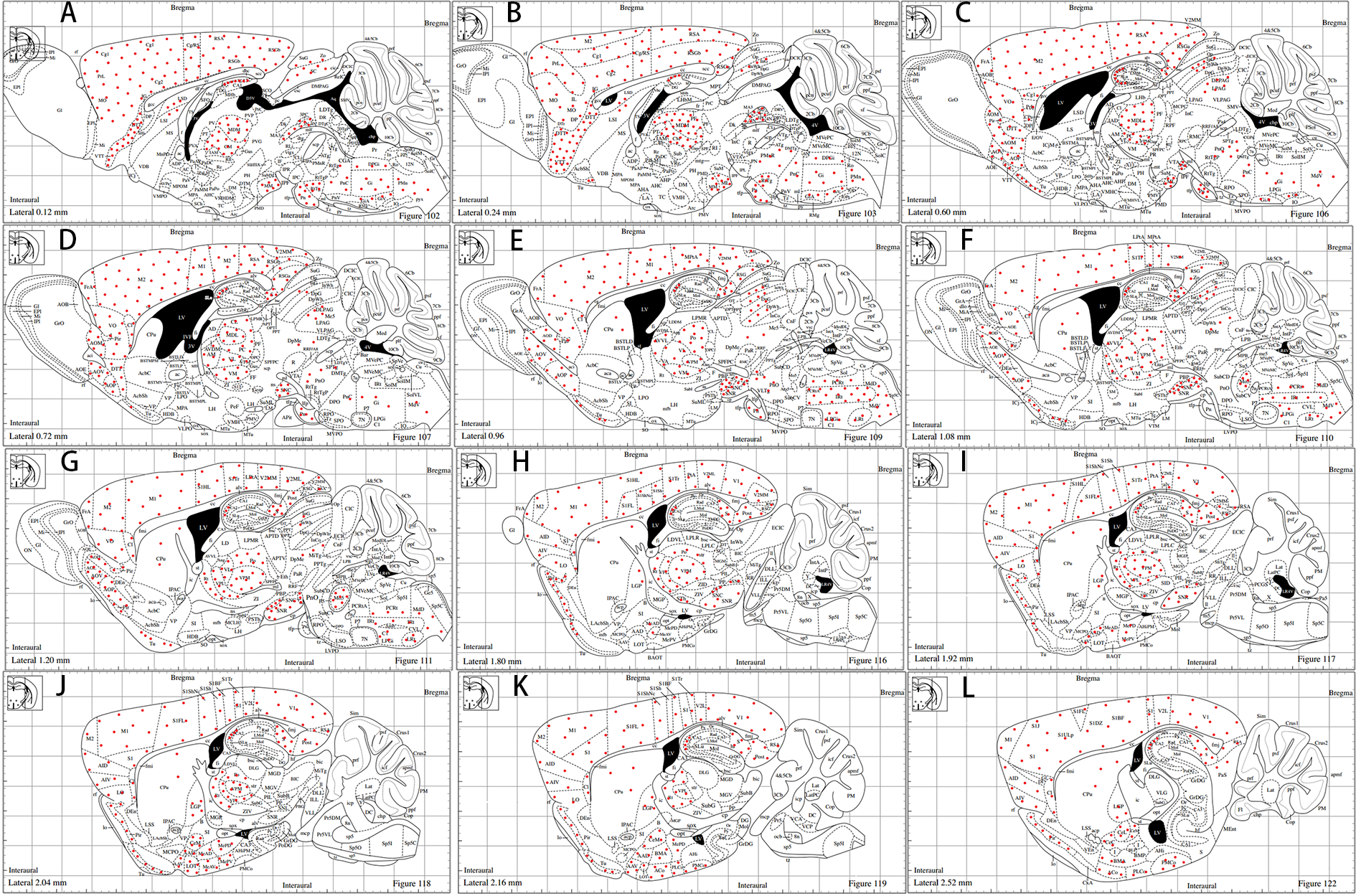

Supplement: S12 Fig — Plotted according to the reference literature [11], the red origin point represents the location of JEV distribution. (A-L) Sagittal images of the brain at a distance of 0.12, 0.24, 0.60, 0.72, 0.96, 1.08, 1.20, 1.80, 1.92, 2.04, 2.16, and 2.52 mm from the mid-sagittal plane, respectively. (TIF) [file pntd.0008442.s012.tif]

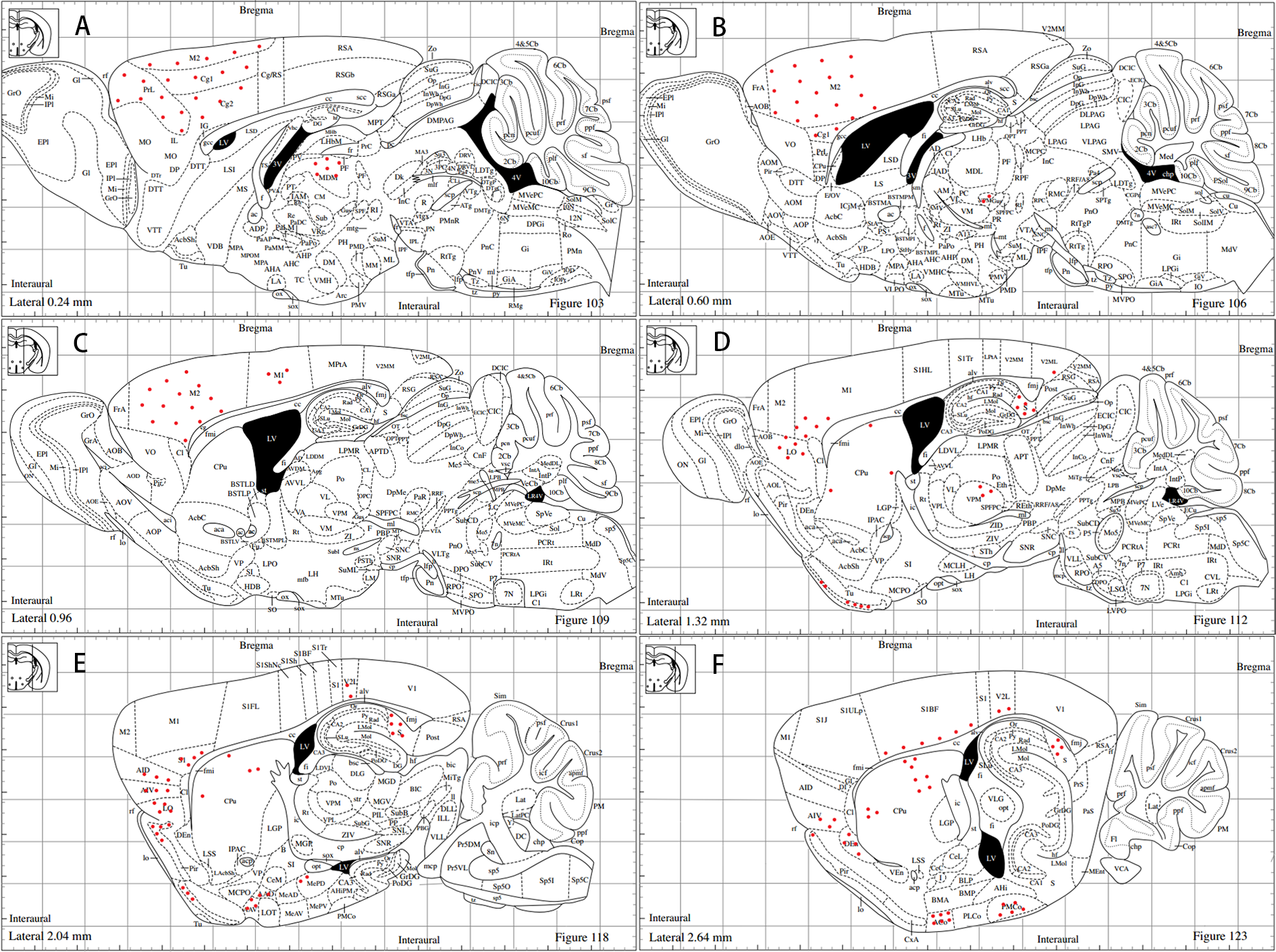

Supplement: S13 Fig — Plotted according to the reference literature [11], the red origin point represents the location of JEV distribution. (A-F) Sagittal images of the brain at a distance of 0.24, 0.60, 0.96, 1.32, 2.04, and 2.64 mm from the mid-sagittal plane, respectively. (TIF) [file pntd.0008442.s013.tif]

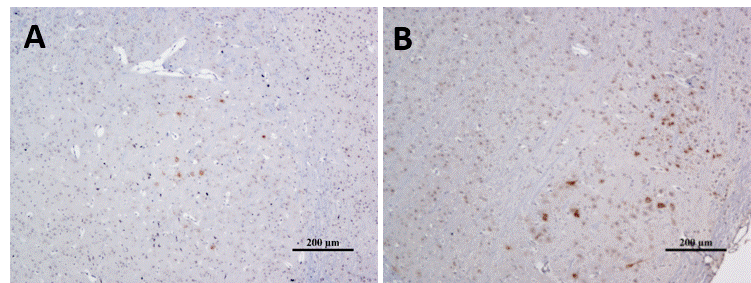

Supplement: S14 Fig — (A) VM. (B) LPGi, LRt. (IHC). (GIF) [file pntd.0008442.s014.gif]

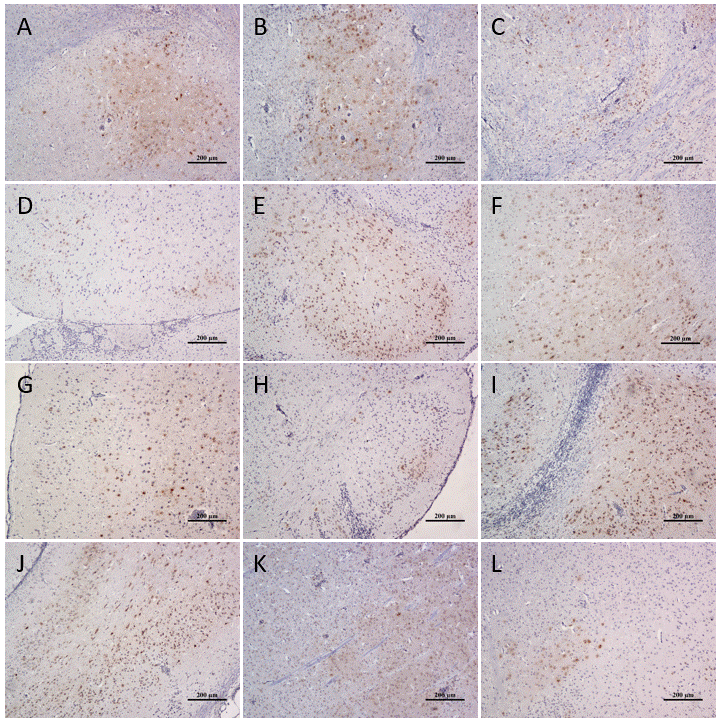

Supplement: S15 Fig — (A) MDM, CM. (B) VL, VM, VPM. (C) VPL, Rt. (D) MO, PrL. (E) DTT, VTT. (F) M1 (G) M2. (H) Tu. (I) AOM, AOV, AOP. (J) AOD, AOL AOV, AOP. (K) CPu. (L) CeL, CeM. (IHC). (GIF) [file pntd.0008442.s015.gif]

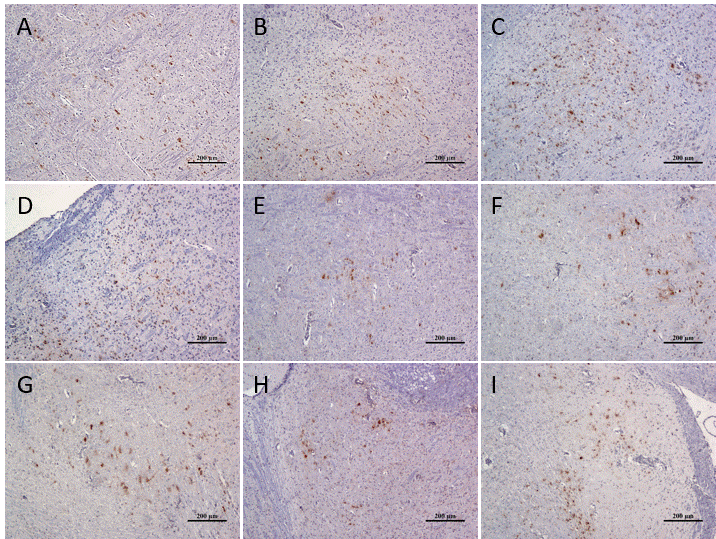

Supplement: S16 Fig — (A) Gi. (B) PMn. (C) PCRt, IRt. (D) MdD. (E) PMnR. (F) PnO, PnC. (G) Mo5. (H) Pr5VL, Pr5DM. (I) SNC, SNR. (IHC). (GIF) [file pntd.0008442.s016.gif]

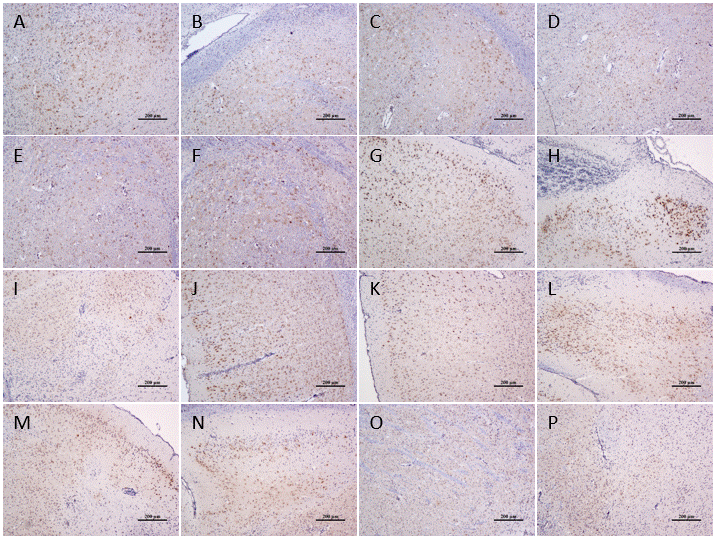

Supplement: S17 Fig — (A) MDM, CM. (B) IAD, AM. (C) VM, VL, PC. (D) CL, MDL. (E) VA, VL. (F) Rt, VPL. (G) PrL, MO. (H) DTT, VTT. (I) AcbC, AcbSh. (J) M1. (K) M2. (L) AOD, AOV, AOP. (M) Tu. (N) Pir, DEn. (O) CPu. (P) CeM, BMP.(IHC). (GIF) [file pntd.0008442.s017.gif]

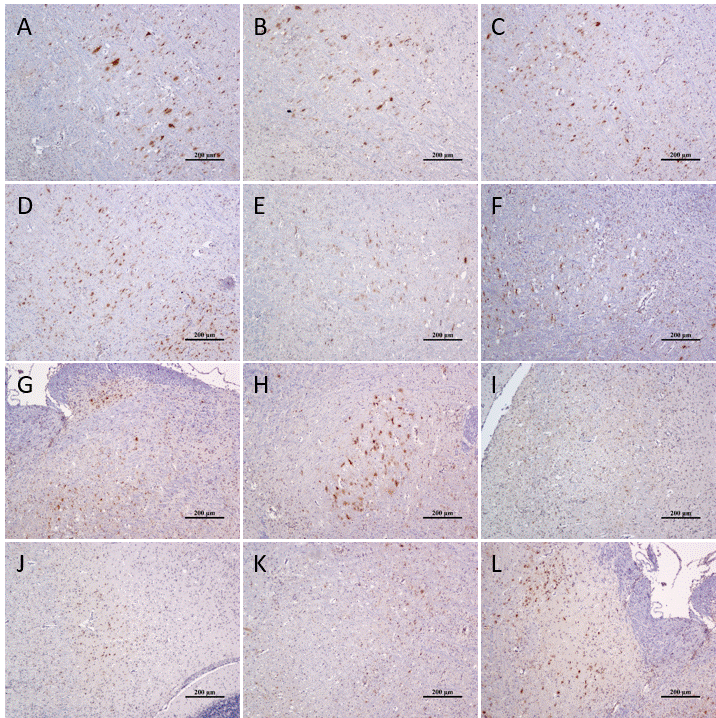

Supplement: S18 Fig — (A) Gi. (B) PMn. (C) PCRt, IRt. (D) MdD, MdV. (E) PMnR. (F) PnO, PnC. (G) VLTg, Pn. (H) Mo5. (I) SuG, SC. (J) DMPAG. (K) DpMe. (L) SNR, SNC. (IHC). (GIF) [file pntd.0008442.s018.gif]

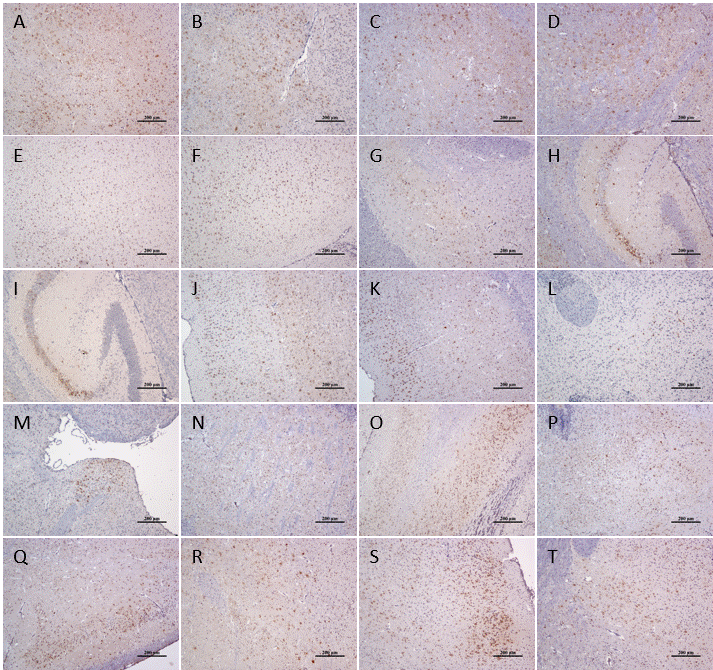

Supplement: S19 Fig — (A) MDM, CM. (B) VL, VM, VPM. (C) Po, VL. (D) Rt, VPL. (E) Cg1. (F) PrL, MO. (G) LSD. (H) CA1. (I) CA2, CA3, Py. (J) M2. (K) M1. (L) LPO. (M) ML. (N) CPu. (O) AOV, AOP, AOD. (P) AcbC, AcbSh. (Q) Pir, DEn. (R) AAD、MeAD. (S) ACo, PLCo (T) CeM. (IHC). (GIF) [file pntd.0008442.s019.gif]

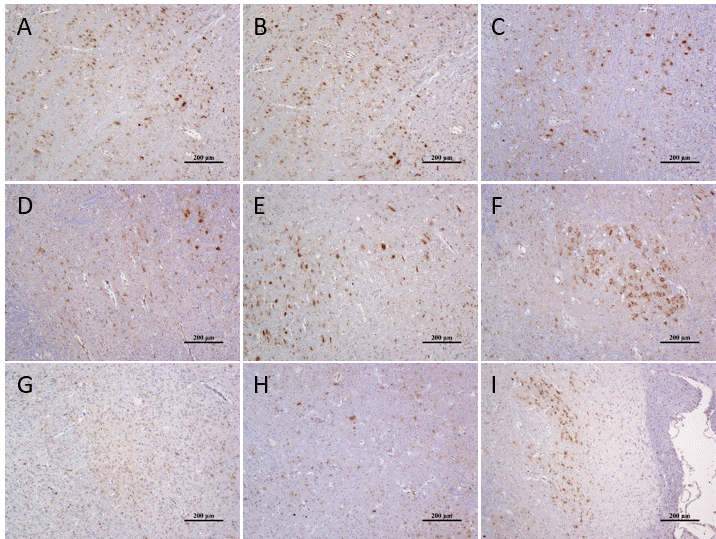

Supplement: S20 Fig — (A) Gi. (B) MdV. (C) PCRt, IRt. (D) PMnR. (E) PnO, PnC. (F) Mo5. (G) DMPAG. (H) DpMe. (I) SNC, SNR. (IHC). (GIF) [file pntd.0008442.s020.gif]

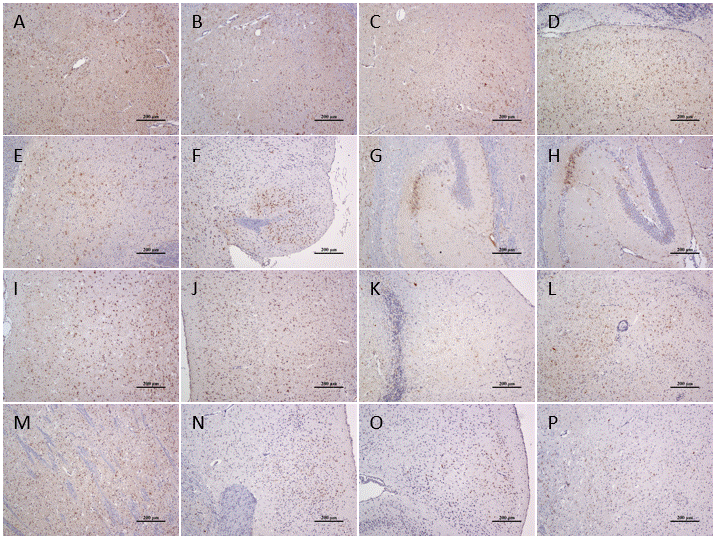

Supplement: S21 Fig — (A) MDM, CM. (B) CL, MDL. (C) VL, VM, VPM. (D) PrL, MO. (E) LSD, LSI. (F) MM, ML. (G) CA1. (H) CA2. (I) M2. (J) M1. (K) AOP. (L) AOP. (M) CPu. (N) MePD, MePV. (O) ACo. (P) CeL, CeM. (IHC). (GIF) [file pntd.0008442.s021.gif]

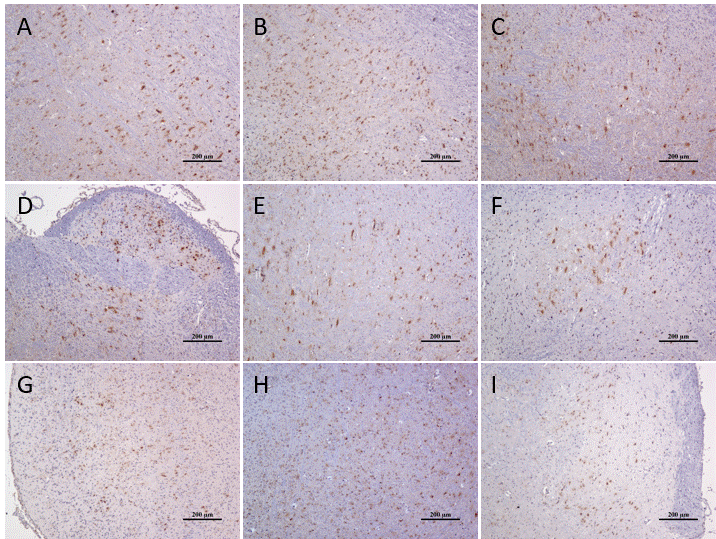

Supplement: S22 Fig — (A) Gi, DPGi. (B) PMn. (C) PMnR. (D) Pn, RtTg. (E) PnO, PnC. (F) Mo5. (G) InWh、DpWh. (H) DpMe. (I) SNC, SNR. (IHC). (GIF) [file pntd.0008442.s022.gif]

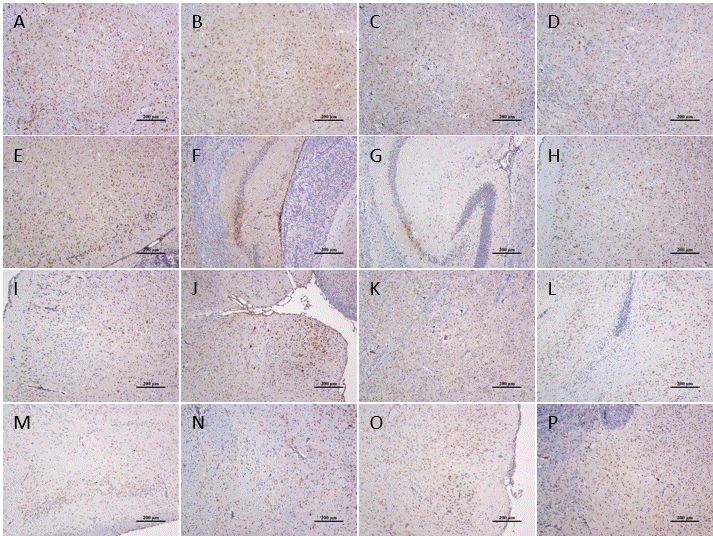

Supplement: S23 Fig — (A) MDM, CM. (B) MDL, PC. (C) VL, VM, VPM. (D) Rt, VPL. (E) PrL, MO. (F) CA1. (G) CA2. (H) M2. (I) M1. (J) MM, ML. (K) CPu. (L) AOM, AOV, AOP. (M) Pir. (N) AAV, AAD. (O) ACo, PLCo. (P) CeM. (IHC). (GIF) [file pntd.0008442.s023.gif]

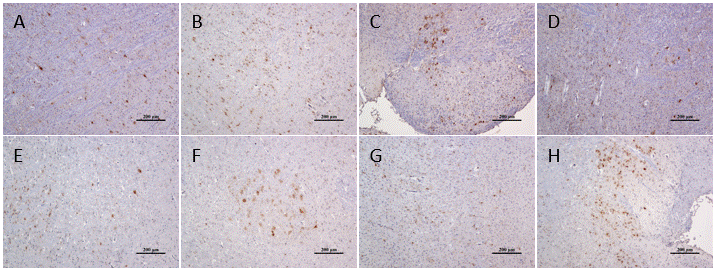

Supplement: S24 Fig — (A) Gi, DPGi. (B) PCRt, IRt. (C) Pn, RtTg. (D) PMnR. (E) PnO, PnC. (F) Mo5. (G) SuG, SC. (H) SNC, SNR. (IHC). (GIF) [file pntd.0008442.s024.gif]

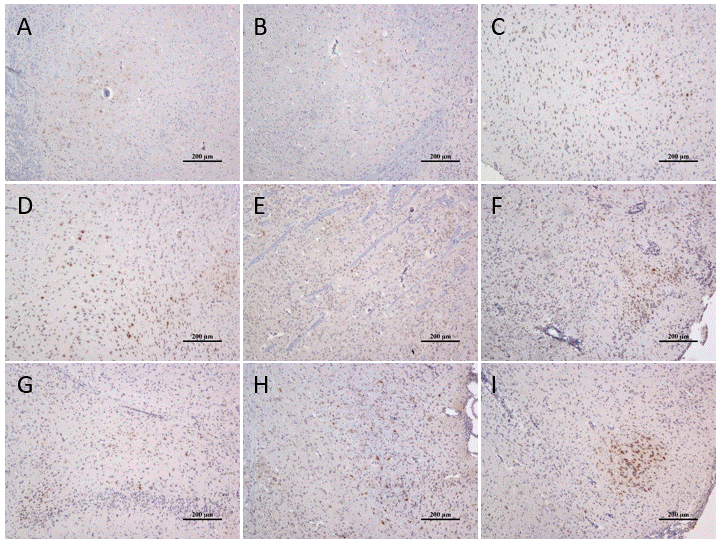

Supplement: S25 Fig — (A) MDM. (B) VPM. (C) PrL. (D) M2. (E) CPu. (F) Tu. (G) Pir, DEn. (H) AAD, AAV. (I) ACo. (IHC). (GIF) [file pntd.0008442.s025.gif]
