# Supplementary material for: Precise localization and dynamic distribution of Japanese encephalitis virus in the rain nuclei of infected mice
Source: PLoS Negl Trop Dis. 2021 Jun 21;15(6):e0008442. doi: 10.1371/journal.pntd.0008442 (PMC8216507; doi:10.1371/journal.pntd.0008442)
Supplement: S1 Table — (DOCX) [file pntd.0008442.s026.docx]

# S1 Table Abbreviations

| **Full name** | **Abbreviation** |
| --- | --- |
| Anterior amygdaloid area, dorsal part | AAD |
| Accumbens nucleus, core | AcbC |
| Accumbens nucleus, shell | AcbSh |
| Anterior cortical amygdaloid nucleus | ACo |
| Anteromedial thalamic nucleus | AM |
| Anterior olfactory nucleus, dorsal part | AOD |
| Anterior olfactory nucleus, external part | AOE |
| Anterior olfactory nucleus, lateral part | AOL |
| Anterior olfactory nucleus, medial part | AOM |
| Anterior olfactory nucleus, posterior part | AOP |
| Anterior olfactory nucleus, ventral part | AOV |
| Field CA1 of hippocampus | CA1 |
| Field CA2 of hippocampus | CA2 |
| Central amygdaloid nucleus, medial division | CeM |
| Cingulate cortex, area 1 | Cg1 |
| Centrolateral thalamic nucleus | CL |
| Central medial thalamic nucleus | CM |
| Caudate putamen (striatum) | CPu |
| Dorsal endopiriform nucleus | DEn |
| Dorsolateral periaqueductal gray | DLPAG |
| Dorsomedial periaqueductal gray | DMPAG |
| Dorsal paragigantocellular nucleus | DPGi |
| Deep mesencephalic nucleus | DpMe |
| Dorsal tenia tecta | DTT |
| Frontal association cortex | FrA |
| Gigantocellular reticular nucleus | Gi |
| Intermediate reticular nucleus | IRt |
| Lateral globus pallidus | LGP |
| Lateral orbital cortex | LO |
| Lateral septal nucleus, dorsal part | LSD |
| Lateral septal nucleus, intermediate part | LSI |
| Primary motor cortex | M1 |
| Secondary motor cortex | M2 |
| Medullary reticular nucleus, dorsal part | MdD |
| Mediodorsal thalamic nucleus, lateral part | MDL |
| Mediodorsal thalamic nucleus, medial part | MDM |
| Medullary reticular nucleus, ventral part | MdV |
| Medial amygdaloid nucleus, anterior dorsal | MeAD |
| Medial mammillary nucleus, lateral part | ML |
| Medial orbital cortex | MO |
| Motor trigeminal nucleus | Mo5 |
| Paracentral thalamic nucleus | PC |
| Parvicellular reticular nucleus | PCRt |
| Piriform cortex | Pir |
| Posterolateral cortical amygdaloid nucleus (C2) | PLCo |
| Paramedian reticular nucleus | PMn |
| Paramedian raphe nucleus | PMnR |
| Pontine reticular nucleus, caudal part | PnC |
| Pontine reticular nucleus, oral part | PnO |
| Posterior thalamic nuclear group | Po |
| Prelimbic cortex | PrL |
| pyramidal cell layer of the hippocampus | Py |
| Retrosplenial agranular cortex | RSA |
| Retrosplenial granular b cortex | RSGb |
| Reticular thalamic nucleus | Rt |
| Primary somatosensory cortex | S1 |
| Primary somatosensory cortex, Forelimb region | S1FL |
| Primary somatosensory cortex, hindlimb region | S1HL |
| Primary somatosensory cortex, trunk region | S1Tr |
| Superior colliculus | SC |
| Substantia nigra, compact part | SNC |
| Substantia nigra, reticular part | SNR |
| Olfactory tubercle | Tu |
| Primary visual cortex | V1 |
| Secondary visual cortex, mediomedial area | V2MM |
| Ventrolateral thalamic nucleus | VL |
| Ventromedial thalamic nucleus | VM |
| Ventral orbital cortex | VO |
| Ventral posterolateral thalamic nucleus | VPL |
| Ventral posteromedial thalamic nucleus | VPM |
| Ventral tenia tecta | VTT |
