# Supplementary material for: Precise localization and dynamic distribution of Japanese encephalitis virus in the rain nuclei of infected mice
Source: PLoS Negl Trop Dis. 2021 Jun 21;15(6):e0008442. doi: 10.1371/journal.pntd.0008442 (PMC8216507; doi:10.1371/journal.pntd.0008442)
Supplement: Attachment — Submitted filename: response.docx [file pntd.0008442.s027.docx]

Dear Editors,

Thank you very much for your coordinating work for my manuscript, and thank the reviewers for their serious guidance and corrections. We have made serious amendments in accordance with the reviewers’ opinions and suggestions. If there are any improprieties that need to be corrected, please let us know.

Sincerely

Changqin GU

Our opinions on reviewers are as follows.

**Methods**

Reviewer #1: Meaning of P3 in the abstract isn’t clear. I suggest changing it to “low passage” and also clearly writing this in the methods. Can they provide the source of the virus as animal or human since this is a key point of the paper.
The paper uses a non-commercial antibody for detection of JEV and it is not written how that antibody was generated. The authors just say “anti-JEV” but it should be more specific what the antigen was and the level of purification of that antigen. A single protein? Purified virus? Etc..

Response#1: JEV P3 strain was isolated from human in China, and its details introduced in GenBank accession no. U47032.1. Please see line378.

Mouse monoclonal antibodies against JEV E proteins were provided by the State Key Laboratory of Agricultural Microorganisms. Firstly, mice are immunized with the JEV SA14-14-2 vaccine strain to prepare monoclonal antibodies. Then, JEV P3 virus and prokaryotic expression E protein are used to screen antibody-positive cell lines for large-scale production of monoclonal antibodies. This method is described in the reference (Shao Lin. Preparation and characteristic analysis of monoclonal antibodies against Japanese encephalitis virus NS5 and E protein[D]. Huazhong Agricultural University, 2013.)

Reviewer #2: (No Response)

Reviewer #3: The study was carried out to localize the JEV in the CNS of mice at a different times post infection, which will be helpful to elucidate the pathogenesis and distribution of the virus in the host. The study design is partly addressed the stated objectives. The sample size is sufficient to ensure adequate power to address the hypothesis being tested. Correct statistical analysis were used. There are concers about ethical requirements being met.

Response#3: Animal experiments in this study was approved by The Scientific Ethic Committee of Huazhong Agriculutral University.

**Results**

Reviewer #1: Figure 1d, each of the features, “glial cell proliferation” “neuronal degeneration” and “necrosis” should be indicated with their own symbols or arrowheads, as is it is not clear. For e, arrows also needed. The aarows are quite small and it would be easier to see if inset images are used. Figures 1-2 could be combined. Control, uninfected tissue is needed.
Fig. 3 legend is out of order, making it difficult to follow. Colocalization cannot be determined from this since it is clearly used on different sections. Staining in similar regions, perhaps, but a technique like confocal is needed to confirm colocalization.
Figure 4 is also not clear how the calculations are being made. Are they based on images similar to those presented in Figure 3? Acronyms in the Fig. 4 legend make it inaccessible. Figures 5-6 legends don’t seem to match. I don’t see any statistics in Fig 6. The image provided in Fig. 6c is not informative.
Much of the information in the supplemental materials should be included in in main figures, particularly those parts describing the course of infection, symptoms, and virus levels.

Response#1: In order to determine the accuracy of the positioning of JEV in the nerve nucleus in this study, some nerve nuclei were verified according to the location of the map. We performed IHC staining with anti-tyrosine hydroxylase polyclonal antibody to locate tyrosine hydroxylase. The tyrosine hydroxylase positive area shows the position of the substantia nigra and striatum (Figure 3B, E). At the same time, HE staining (Figure 3A, D) and JEV immunohistochemical staining (Figure 3C, F) were performed on serial sections. According to the mouse brain anatomy atlas, the presence of JEV antigens in the substantia nigra and striatum was determined 96h after infection. We showed the pictures to verify the accuracy of JEV positioning in the area of the map, not colocalization in one cell.

For Figure 4, we calculated based on Figure 5A. The scanned digital sections were analyzed using Aperio ImageScope software, and the positive rate of JEV in the major nerve nuclei was measured according to the atlas location (3-5 images per nucleus were counted to calculate the percentage of the optical density of the virus-positive signal, and the nerve nucleus area).

Regarding the last few pictures, I am very sorry, because we put the serial number wrong, which caused your misunderstanding.Now we change fig4 to fig5, fig5 to fig6, and fig6 to fig4.Please see line 259-291.

Reviewer #2: (No Response)

Reviewer #3: The analysis presented match the analysis plan. The results are clearly and completely presented. The figures are sufficient quality for clarity

**Conclusions**

Reviewer #1: It should be discussed that this strain causes a non-lethal infection, unlike the Nakayama strain which is well characterized and produces lethal disease in mice. In general, the authors do not cite enough studies that describe histology/pathology of mouse brains in experimental JEV infection.？？
As mentioned elsewhere, the conclusions of colocalization are weak and not supported by techniques that would allow those statements.

Response #1: Thank you for your valuable comments. In this study, the infected mice had a fatality rate of 40%. Of course, we also observe the clinical symptoms and pathological changes of the brain tissue of the mice during the whole experiment. Mice usually die 5-7 days after infection. Mice infected after 2 weeks may be tolerant. Many scholars have done these studies, so there is not much explanation. Because we want to focus on observing the dynamic distribution of the virus in the brain of mice after infection, we did not describe the pathological changes in detail, but it can be seen that the amount of virus in the mouse brain is related to the severity of the pathology.

Reviewer #2: (No Response)

Reviewer #3: The conclusions are supported by the data presented. The authors discuss that these data explains the relationship between JEV infected nerve nuclei and the clinial symptoms. No public health relevance is addressed.

Response #3：Thanks for your comments.

Summary and General Comments

Reviewer #1: This manuscript uses a non-lethal strain of JEV and identifies areas of infection in the brain of experimentally infected mice. This is a study to describe the regions of the brain where JEV is detected. An emphasis is placed on the localization of JEV to “nerve” nuclei, but there is insufficient dual-staining with markers to identify the localization of JEV at a cellular level.  The manuscript order made it very difficult to review. There also appear to be some major issues with the matching of figure legends to figures that make the manuscript impossible to fully assess in the current format. The quality needs to be improved significantly before this would be ready for in-depth review and publication.

Response #1: Thank you for your careful review of our paper and your valuable comments. The study examined the dynamic positioning of JEV in the brain's nuclei after JEV infection. I'm very sorry for the trouble caused by the wrong sequence number of the pictures. We will further improve and modify according to your comments and suggestions.

Reviewer #2: This manuscript by Han and colleagues describes a Japanese encephalitis virus (JEV) neuropathology model in mice.  The longitudinal study design allowed the investigators to examine JEV antigen distribution and pathology at different times post infection.  While nothing particularly novel was discovered in this study that has not been described previously for JEV or other neurotropic flaviviruses, the work appears to be well done, is very detailed, and supports observations described in the literature.  Furthermore, the longitudinal brain sampling permits some inferences as to the mode of entry of JEV into the central nervous system (CNS).  More specific comments are below.

**Major comments:**

1. While the intraperitoneal (IP) route of inoculation is an acceptable way to challenge mice with JEV, it does not really mimic natural infection.  It is possible that virus replication kinetics post IP inoculation vary greatly from more natural routes of exposure such as intradermal (ID).  The route of inoculation could also impact the mode of virus neuroinvasion.  This is a major limitation of this mouse model.  Did the authors consider using ID inoculation?  Furthermore, can the authors comment on the use of a 10^6 PFU challenge dose (line 367 of the manuscript).  This dose is quite high and could also impact the factors listed above.

Response 1: Thanks to the reviewers for their suggestions, the different injection methods and their results need to be studied. In the study of the pathogenicity of neurotropic viruses, the brain challenge can reflect the neurovirulence of the virus, and the result of the peripheral challenge reflects the neurovirulence and neuroinvasiveness of the virus. Our idea is that through peripheral vaccination, JEV can break through the blood-brain barrier and reach the central nervous system. The intraperitoneal injection method meets our needs. In the preliminary experiment, we determined the challenge dose of 10^6^. This dose ensures that JEV can successfully invade the central nervous system without causing excessive mortality in mice, ensuring the collection of subsequent test samples. In the research of Ye Jing et al, mice were injected intraperitoneally with 10^6^ JEV, and mice showed obvious neurological symptoms at day 6 after infection.
2. Line 39, what is meant by “consistent concentration”.  Was a concentration measured?

Response 2：Thank for your suggestion，we reversed the sentence to “and the virus content is relatively stable”. Please see line 39.

3. Throughout the manuscript, hours post infection are reported.  Consider also indicating the number of days in parentheses as many researchers are accustomed to seeing time post infection represented this way.

Response 3： Thanks for your suggestions. We have been modified all the time units in the manuscript.
4. Line 82, mice are “a good” model for JEV, I would not consider them to be “an ideal”, as stated here.  Please consider revising.

Response 4: Thanks for your advice. We have changed “a good animal model” to “an ideal animal model”. Please see line 82.

5. Throughout the manuscript, change “symptoms” to “signs” when referring to mouse studies.

Response 4: Thanks for your suggestion. We have changed “symptoms” to “signs”. The text is highlighted with a yellow background.

6. Line 91, consider changing “fluffy coat” to “ruffled fur”, which is more commonly used.  Also, what is meant by “curling behavior”?  Are the authors referring to “hunched posture”?

Response 4: Thanks， We have changed “fluffy coat” to “ruffled fur”, and changed “curling behavior” to “hunched back” according your advance. Please see line 91.
7. The paragraph starting on line 176 contains sentences with very long lists of brain regions and it is very difficult to read.  Can a Table/Figure be referenced and a very general statement be made in the text instead of listing everything.

Response 7: Thanks for your good suggestions. We tried to make a table to describe the results. Because there are too many nerve nuclei, the table was too complicated to be eventually abandoned.

8. Figure 5 and 6 legends do not match with the actual Figures.

Response 8:Yes, the order of the legends in the text is wrong. We have changed fig4 to fig5, fig5 to fig6, and fig6 to fig4.

9. Line 308, was the “viral load” in the brain measured?  Usually this refers to infectious virus or viral genome copy measurements.  Consider rewording.

Response 9: Thanks for your reasonable suggestions. We have change “viral load” to “positive signal of JEV”. Please see line 327.

10. Line 396, is the “anti-JEV primary antibody” mouse antiserum?  How was it generated?  Please indicate in the manuscript.

Response 10： Yes，mouse monoclonal antibodies against JEV E proteins were provided by the State Key Laboratory of Agricultural Microorganisms. We have added the details in the method. Please see line 417-424.

Reviewer #3: In this article, Wei Han et al established a mouse model infected with Japanese encephalitis virus (JEV). The virus dynamical localization and the distribution were depicted through the IHC and ISH and construction of three-dimensional (3D) images. The results showed that the viral localization and distribution changed in a time-dependent manner and consistent with the clinical symptoms. Although the detailed dynamic localization and distribution will be helpful to know the virus entry pathway and transmission, it seemed very weak and ineffective for clarifying the pathogenesis of the virus. No clear innovation was discovered for the transmissible mechanism of the disease.  It may suggest the authors should localization how the virus passes through the blood-brain barrier.

Response3: Thanks for the reviewer’s suggestion. It is true that many studies have shown that JEV has several ways to enter the brain tissue, and the main research focuses on the blood-brain barrier. This study did not pay attention to how the virus enters the brain tissue, but once the virus is found in the brain, the number of viruses may increase rapidly. And the nerve nucleus of the virus first discovered in the brain, such as the AO nucleus, may provide some help for future research on how the virus enters the blood-brain barrier. we are also very interested in it, then further research is needed.
