# Supplementary material for: Precise localization and dynamic distribution of Japanese encephalitis virus in the rain nuclei of infected mice
Source: PLoS Negl Trop Dis. 2021 Jun 21;15(6):e0008442. doi: 10.1371/journal.pntd.0008442 (PMC8216507; doi:10.1371/journal.pntd.0008442)
Supplement: Attachment — Submitted filename: revision letter.docx [file pntd.0008442.s029.docx]

**Methods**

Reviewer #3: (No Response)

Reviewer #4: The methods are clearly explained. However, some edits are needed.

1) Line 372- not sure what the sentence means.

Response 1): We have modified this sentence, which means Hubei Provincial Laboratory Animal Quality Certificate Number. Please see lines 462 and 484
2) Please refrain from describing euthanasia methods and delete lines 373-374.
Response 2): Description of euthanasia method have been deleted from the text. Please see line 486.

3) Is 2 days enough to inactivate JEV? (line 376).

Response 3): The role of formaldehyde fixation makes the protein coagulate and avoids the disappearance or dispersion of the antigen in the tissue or cell. 2 days fixing for half the brain to inactivate viruses is enough. (line 488)

**Results**

Reviewer #3: (No Response)

Reviewer #4: The results are described clearly. However, considerable editing is required.

1) Animals do not exhibit symptoms but rather signs. Please replace "symptoms" with "signs".
Response 1): All “symptoms” in the text were replaced with “signs ” . Please see lins 29, 30, 100,112 and so on.

2) Please do not state that "animals died (Line 95). Every approved animal protocol has euthanasia criteria. Please replace "died" with "euthanized".

Response 2): The word ”died” was replaced with the word “euthanized” in line 117.

**Conclusions**

Reviewer #3: (No Response)
Reviewer #4: The conclusions as well supported and stated clearly.

**Editorial and Data Presentation Modifications**

Reviewer #3: (No Response)
Reviewer #4: Please hyphenate after "post" throughout the manuscript i.e. post-infection.
Response：All “post infection” were replaced with “post-infection”.
Please abbreviate "hours post-infection (hpi)" and use "hpi throughout the manuscript.

Response: We have changed “hours post-infection (hpi)” to “day post-infection ”according to the first revision. We abbreviate “day post-infection (dpi)"and use "dpi throughout the manuscript for this revision. Please see lins 30, 32, 33 and so on.

Please italicize "in situ" throughout the manuscript.

Response：we changed the word “in situ” into *“in situ*” in line 26,168,170,311,494,517.
Please add white space in figure 1 and 2 to distinguish each picture.

Response: We added white space in figure 1 and 2.

**Summary and General Comments**

1. Lines 160-162, 226-227: Missing space around parentheses.

Response: The spaces were added around parentheses. please see in line 196-199,277.
2. Page 2, line 32: “which lasted up to 、8d” should be corrected to“which lasted up to 8d”
Response: The phrase “which lasted up to 、8d” was corrected to “which lasted up to 8d”. please see in Page 2, line 31.

3. Page 17, line 362：“……21 day post treatment” should be corrected to “……21 day post infection”.
Response: “……21 day post treatment” was corrected to “……21 day post infection”. please see in Page 20, line 466.

4. It’s difficult to understand statistical analysis in figures of Fig. 4. Were all groups compared with IRt? It’s suggested that the significant differences among groups should be illustrated in the legend or marked in the figures.

Response: Thank you for your suggestion, we have illustrated in the legend to indicate the significant differences between the groups. please see in line 327-329.

5. The legend of figure F in Fig.2 was missing.
Response: We modified the legend. Please see line 311.

6. The revision in line 39 was not found. Reviewer #2: Line 39, what is meant by “consistent concentration”. Was a concentration measured? And the response 2：Thank for your suggestion，we reversed the sentence to“and the virus content is relatively stable”. Please see line 39.
Response: Sorry for my fault. We reversed the sentence to “and the virus content is relatively stable”. Please see line 38.

7. The revision in line 82 was not found. Reviewer #2: Line 82, mice are “a good” model for JEV, I would not consider them to be “an ideal”, as stated here. Please consider revising. And the response 4: Thanks for your advice. We have changed “a good animal model” to “an ideal animal model”. Please see line 82.
Response: We have changed “a good animal model” to “an ideal animal model”. Please see line 101.

8. The revision in line 91 was not found. Reviewer #2: consider changing “fluffy coat” to “ruffled fur”
Response: We have changed “fluffy coat” to “ruffled fur”. Please see line 114.

9. The revision in line 327 was not found. Response 9: Thanks for your reasonable suggestions. We have change“viral load” to “positive signal of JEV”. Please see line 327.
Response: We have changed “viral load” to “positive signal of JEV ”. Please see line 421.

10. The revision in line 417-424 was not found. Response 10： Yes，mouse monoclonal antibodies against JEV E proteins were provided by the State Key Laboratory of Agricultural Microorganisms. We have added the details in the method. Please see line 417-424.
Response: We have added the details in the method. Please see line 504-509.
